# Systematic Exploration of the Association Between Vitamin A Intakes and Sarcopenia Prevalence in American Adults

**DOI:** 10.1002/fsn3.4613

**Published:** 2024-11-25

**Authors:** Meisi Fang, Jinghang Ma, Yongyu Bai, Yifan Ying, Xian Shen, Zhen Feng

**Affiliations:** ^1^ School of the First Clinical Medical Sciences (School of Information and Engineering) Wenzhou Medical University Wenzhou China; ^2^ Department of Gastrointestinal Surgery the First Affiliated Hospital of Wenzhou Medical University Wenzhou China; ^3^ Information Technology Center Wenzhou Medical University Wenzhou China

**Keywords:** dietary vitamin A, gender‐specific association, mediation analysis, mixture analysis, muscle mass, sarcopenia

## Abstract

Sarcopenia, characterized by an age‐related progressive loss of muscle mass and strength, presents significant health concerns. Recommending dietary nutrition emerges as a viable strategy to counteract muscle deterioration. Vitamin A, indispensable throughout the human life cycle and unattainable through endogenous synthesis, necessitates intake via diet. However, the direct correlation between sarcopenia prevalence and vitamin A intake remains unclear. This study systematically investigated the relationship between sarcopenia prevalence and vitamin A intake, including retinol and some carotenoids, across diverse races and genders utilizing multiple statistical analyses. Mixture analysis revealed significant positive correlations between total vitamin A intake and muscle mass among American adult males (Male: OR: 1.019, 95% CI: 1.010–1.027, *p* < 0.001). We also observed the gender‐specific results, with retinol playing a more significant role in enhancing muscle mass for males, while certain carotenoids were found to be more influential in females. Moreover, inflammation and oxidative stress mediated the relationship between vitamin A intake and sarcopenia prevalence in both genders. There may be a gender‐ and race‐specific relationship between dietary vitamin A intake and sarcopenia. Further prospective studies are imperative to elucidate the association between vitamin A intake and sarcopenia prevalence.

## Graphical abstract

This study systematically explores the association between dietary vitamin A intake and sarcopenia prevalence among American adults, highlighting significant gender‐ and race‐specific relationships. Our findings indicate that higher total vitamin A intake is positively correlated with muscle mass in males, while specific carotenoids play a more prominent role in females, with inflammation and oxidative stress mediating these effects. Further research is needed to clarify these associations and their implications for dietary recommendations.

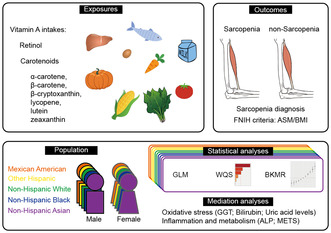

## 1 Introduction

Sarcopenia, a geriatric syndrome marked by the gradual decline in both skeletal muscle mass and function, represents a significant health concern, particularly among aging populations (Rosenberg 1997). As a multifaceted condition, sarcopenia not only contributes to diminished physical performance but also poses a substantial risk for adverse health outcomes, including falls, fractures, and a decline in overall quality of life (Cruz‐Jentoft and Sayer 2019). Recommending dietary nutrition may be an appropriate approach to combat muscle deterioration.

Vitamin A, the first vitamin to be discovered (Tee 1992), is an essential fat‐soluble vitamin throughout the human life cycle and cannot be synthesized by the body, necessitating intake through the diet (Trumbo et al. 2001). Depending on the source, vitamin A can be classified into two categories. Animal‐derived sources such as eggs, milk, and liver mainly provide retinol, which regulates various developmental and metabolic processes in vivo. Fruits and vegetables are rich in provitamin A carotenoids, such as beta‐carotene, alpha‐carotene, and beta‐cryptoxanthin. These carotenoids have the ability to be transformed into retinol in the intestines. Nevertheless, the majority of carotenoids present in these foods are non‐provitamin A types, including lutein, zeaxanthin, and lycopene, which serve other beneficial functions in the body (Asgari, Brasky, and White 2012).

Vitamin A holds a pivotal position in cell growth and ontogeny, and recent inquiries have delved into its potential impacts on muscle development. Experimental evidence has shown that vitamin A stimulates the differentiation of muscle cells and facilitates the repair of skeletal muscles, indicating its significant role in maintaining muscle health (Zhang et al. 2023). Furthermore, administering vitamin A injections to newborn calves enhances the expression of crucial myogenic genes and proteins, thereby fostering the development of calf muscles. Additionally, carotenoids, which are abundant in carotenoid‐rich foods, have been linked to preventing the decline in muscle strength and walking difficulties among elderly individuals (Wang et al. 2018). However, the direct association between the prevalence of sarcopenia and vitamin A intake remains unclear.

Researches have indicated that both the prevalence of sarcopenia and vitamin A intake are influenced by differences in sex and race. The pathogenesis of sarcopenia involves a multifactorial process, with the impact of androgens, estrogen, and progesterone on sarcopenia varying according to sex (Kim et al. 2016; Knapik and Hoedebecke 2021). Differences in lean body mass, muscle strength, and physical capabilities are apparent among various racial groups (Goodpaster et al. 2006). Dietary assessments unveil variations in the average levels of retinol equivalents across genders (Garry et al. 1987), and a single 24‐h dietary recall indicated differences in dietary vitamin A intake among diverse races in American adults (Cheng et al. 2023).

Moreover, numerous studies have shown that inflammation and oxidative stress play a role in the initiation of sarcopenia. Vitamin A is considered an antioxidant molecule, that could protect lipids against rancidity (Burton and Ingold 1984; Finaud, Lac, and Filaire 2006; Petiz et al. 2017). Dietary carotenoids and retinoids have been established as crucial players in innate and acquired immunity, as well as in the body's inflammatory response mechanisms (Rubin et al. 2017). Considering the above findings, we hypothesized that oxidative stress might mediate the link between vitamin A and sarcopenia prevalence, offering valuable insights into the underlying mechanisms.

In this study, we systematically explored the association between vitamin A intake and sarcopenia prevalence across diverse ethnicities and genders, employing the generalized logistic model (GLM) and two advanced mixture analysis methods. We further conducted a mediation analysis to investigate the potential mediating effects of inflammation, oxidative stress, or other factors in the association between vitamin A intake and sarcopenia prevalence.

## 2 Methods

### 2.1 Study Population

The National Health and Nutrition Examination Survey (NHANES) is a comprehensive, population‐based, cross‐sectional study that aims to gather extensive data on the health and nutritional statuses of adults and children living in the United States. This valuable resource is provided free of charge by the Centers for Disease Control and Prevention's National Center for Health Statistics (NCHS) and is accessible online at https://www.cdc.gov/nchs/. We analyzed data from the last three periods (2011–2012, 2013–2014, and 2015–2016). During this time, a total of 29,902 participants were enrolled in NHANES for three consecutive survey cycles. In this study, 25,542 participants were excluded for being under 35 years old (as muscle decline typically begins around this age) or lacking data on dietary vitamin A, appendicular skeletal muscle mass (ASM), or body mass index (BMI). And then participants who did not have data on family income‐to‐poverty ratio (PIR), hypertension, diabetes, cholesterol, and protein intake were also excluded. The final study population for this study was 3911 people, of which 1849 were males and 2062 were females.

### 2.2 Evaluation of Retinol and Carotenoid Consumption

Since the early 1970s, NHANES has been consistently gathering dietary intake data through diverse methodologies. The principal endeavor for the collection of dietary data involves initiating a 24‐h recall procedure through NHANES. Also included is a Food Frequency Questionnaire (FFQ) with a different number of questions, the focus of which varies over the course of the survey cycle. The 24‐h recall method is typically employed for assessing dietary intake in extensive surveys. Since 2002, NHANES has been collecting data on food and nutrient intake through two 24‐h recalls, utilizing the standardized USDA automated multiple‐pass methods and databases (Blanton et al. 2006). This approach aims to provide an efficient and accurate method for collecting intake data in large‐scale national surveys (Willett 1998). Moreover, NHANES participants are eligible to undergo two distinct 24‐h dietary recall interviews. The first interview, which involves a face‐to‐face encounter, is conducted at the Mobile Examination Center (MEC). Subsequently, a follow‐up interview is conducted via telephone, typically within a timeframe of 3–10 days following the initial interview. This comprehensive approach ensures a thorough assessment of dietary habits and nutritional intake among NHANES participants (Lin, Lyu, and Feng 2024). Vitamin A was obtained and recorded at baseline as a continuous variable. Total vitamin A is derived from the combined effects of retinol and carotenoids, taking into account their individual vitamin A potencies. Rigorous validation studies involving cohort participants have established the FFQ as a reliable and precise tool for assessing individuals' nutrient intake (Feskanich et al. 1993). In our study, we incorporated retinol and a selection of carotenoids, specifically six distinct carotenoids: alpha‐carotene, beta‐carotene, beta‐cryptoxanthin, lycopene, lutein, and zeaxanthin.

### 2.3 Assessment of Covariates and Mediators

We incorporated clinically meaningful covariates in our study, that could potentially influence the sarcopenia or the concentration of dietary vitamin A, including age (≥ 35 years), race (Mexican American, other‐Hispanic, Non‐Hispanic White, Non‐Hispanic Black, non‐Hispanic Asian, and others), education (less than high school, high school, and college), marital status (married, never married, and others), family poverty income ratio (PIR), body mass index (BMI), weight, arm circumference (AC), waist circumference (WC), and protein intakes. Drinking was classified into two categories (“No”: < 12 drinks per year or drinking frequency ≤ 2 in the past 12 months, “Yes” for the other cases). Smoking status was defined as two categories (“No”: Smoked < 100 cigarettes in their lifetime, “Yes”: Smoked ≥ 100 cigarettes). The presence of hypertension refers to average systolic pressure ≥ 140 mmHg, average diastolic pressure ≥ 90 mmHg, or self‐reported intake of antihypertensive medication. High cholesterol was defined as having total cholesterol levels of ≥ 240 mg/dL or self‐reported intake of prescribed medication for managing cholesterol. The pre‐existing conditions of diabetes disease were determined through self‐reported diagnoses provided by a physician. Furthermore, we delved deeper into the interplay between dietary vitamin A intake and various biological markers such as oxidative stress (measured through GGT, bilirubin, and uric acid levels), inflammation (assessed by ALP), and metabolism (evaluated by METS). Using mediation analyses, we aimed to unpack the direct and indirect relationships, as well as the magnitude of any mediating effects, in this complex web of interactions.

### 2.4 Assessment of Sarcopenia

In the latest iteration of the NHANES, dual‐energy X‐ray absorptiometry (DXA) is utilized for body composition analysis, representing a sophisticated and accurate measurement technique. Specifically, NHANES utilizes the Hologic QDR 4500A fan‐beam bone densitometers in DXA to gather nationally representative data pertaining to bone and soft tissue measurements. This comprehensive approach encompasses measurements of the total body, both arms and legs, the trunk, and the head. DXA has gained widespread acceptance as the preferred method for assessing body composition, attributed to its swiftness, user‐friendliness, and minimal radiation exposure (Heymsfield et al. 1989; Baran et al. 1997). Moreover, comprehensive information regarding the DXA examination protocol is outlined in the Body Composition Procedures Manual, which is accessible on the NHANES website, providing a detailed reference for researchers and healthcare professionals. The metrics that can be derived from DXA include arm and leg lean mass (kg). Actually, the lean mass obtained from DXA also included nonbone as well as nonfat tissue (Cruz‐Jentoft and Sayer 2019). Appendicular skeletal muscle mass (ASM) was determined by aggregating the lean soft‐tissue masses found in the arms and legs, offering a thorough evaluation of the muscular composition in these specific areas (Heymsfield et al. 1990).

In this investigation, the sarcopenia index was derived by normalizing appendicular skeletal muscle mass (ASM) to body mass index (BMI), resulting in ASM/BMI, without considering measurements of muscle strength and function. Participants were categorized as having sarcopenia if their sarcopenia index fell below the threshold values of 0.789 for males and 0.512 for females, respectively. These criteria were established by the recent consensus meeting of the “Foundation for the National Institutes of Health (FNIH) Sarcopenia Project” and have been widely employed in recent research (Studenski et al. 2014).

### 2.5 Statistical Analyses

GLM represents an extension of traditional regression models, specifically tailored to accommodate error distributions that deviate from the normal distribution (Boughorbel, Al‐Ali, and Elkum 2016). In our study, we used GLM analysis to find a relationship between retinol and some carotenoids and sarcopenia. GLMs extend the capabilities of regression models by catering to error distributions that transcend the normal distribution (Boughorbel, Al‐Ali, and Elkum 2016). We started by separating the data by gender. Retinol and some carotenoids were used as continuous variables, which were categorized into four levels using the interquartile method assigning values of 0, 1, 2, and 3, respectively, and a model was constructed using a dummy variable approach with the lowest quartile of retinol and some carotenoids as the reference in our study (Lyu, Wang, and Dong 2023). In addition, the rank variable was subsequently brought into the regression model again, and the resulting *p* value corresponding to the rank variable was the result of the *p* for trend test. The model was adjusted to account for potential confounders, encompassing factors such as age, race, family PIR, diabetes, hypertension, and cholesterol levels.

The weighted quantile sum (WQS) regression, a specific analytic approach tailored for estimating the effects of exposure mixtures, has gained significant popularity as a means to analyze the association between exposure mixtures and health outcomes (Yorita Christensen et al. 2013). The WQS regression model, along with the calculation of the weighted index, proposed by (Carrico et al. 2015) is represented by

$$g\left(\mu \right)=\beta_{0}+\beta_{1}\left(\sum_{i=1}^{N}w_{i}q_{i}\right)+z^{\prime }\phi$$

$$\mathrm{WQS}=\sum_{i=1}^{N}{\bar{w}}_{i}q_{i}$$

where $\beta_{0}$ is the intercept; $z^{\prime }$ and ϕ serves as representations of the matrix of covariates and the coefficient of covariates, respectively;$\;{\bar{w}}_{i}$ embodies the significance of weight for the $i^{\mathrm{th}}$ element component in the $i^{\mathrm{th}}$ group; $q_{i}$ is the quantile of the $i^{\mathrm{th}}$ element in the $i^{\mathrm{th}}$ group; and the summation $\sum_{i=1}^{N}{\bar{w}}_{i}q_{i}$ signifies a weighted index for the group of *N* elements of concern within the specified group i.

Bayesian kernel machine regression (BKMR) represents a sophisticated statistical approach that leverages a kernel function to adaptively capture the intricate individual and interactive effects associated with exposure to complex mixtures of chemicals (Bobb et al. 2015, 2018). It enables the visualization of individualized exposure‐response profiles, taking into account concurrent exposures while accommodating potential non‐linear relationships and/or differential effect directions among various exposures. Consequently, the independent contributions of retinol and six distinct carotenoid components can be discerned by extracting the posterior inclusion probabilities (PIPs), and the overall cumulative impact of the mixed exposure can be captured (Preston et al. 2020). For this approach, we conceptualized the sarcopenia outcome as a smoothly varying function, expressed via a kernel function, that depended on the exposure variables while accounting for potential confounding factors. BKMR model can be represented as follows: For each i=1,…,N,

$$Y_{i}=h\left(z_{i}\right)+x_{i}\beta +e_{i}$$

The function *h* captures the effect of exposure in a high‐dimensional scenario, while *z* represents the collection of multiple exposure variables. The variables x and their coefficients β represent the confounders and their associated weights, respectively. Finally, it is assumed that the residual, denoted as e, follows a normal distribution. The bkmr analyses were conducted using bkmr R Package (version 0.2.2).

### 2.6 Mediation Analyses

Mediation analyses address the question of the mechanism by which an exposure leads to an outcome by expressing the overall exposure effect as a combination of indirect and direct effects (Mackinnon and Fairchild 2009). In general, for mediation analyses to be valid, more confounding factors must be considered than the estimated overall effect size. A regression‐based approach is used to ensure that all relevant confounders are considered in the mediation analysis. This paper performed mediation analyses to examine how dietary vitamin A affected the development of sarcopenia through pathways involving oxidative stress (GGT, bilirubin, and uric acid), inflammation (ALP), or metabolism (METS), respectively.

## 3 Results

### 3.1 Population Characteristics

In our study, an initial cohort of 29,902 participants from NHANES 2011–2016 were enrolled. After excluding individuals younger than 35 years old, as well as those with missing covariates and dietary vitamin A data, the final study population comprised 3911 subjects, including 1849 males and 2062 females. The flowchart in Figure S1 illustrates the process of participant screening and inclusion.

The demographic characteristics of the study cohort are shown in Table 1. The non‐sarcopenia group, in contrast to the sarcopenia group, was characterized by a younger age distribution, higher levels of income, and a majority with university‐level education. Significant differences were found in various socio‐economic and other factors, including age, race, education, family PIR, alcohol consumption, hypertension, diabetes, cholesterol, body mass‐related indicators, and other factors between participants with and without sarcopenia (*p* < 0.001).

**TABLE 1:** Demographic characteristics and sarcopenia outcomes disaggregated derived from NHANES 2011–2016, consisting of 3911 participants.

	Non‐sarcopenia (*n* = 3545)	Sarcopenia (*n* = 366)	*p*
Demographic			
Age, years	47.00 [41.00, 52.00]	50.00 [43.00, 55.00]	< 0.001
Sex, *n* (%)			
Male	1694 (47.8)	155 (42.3)	0.054
Female	1851 (52.2)	211 (57.7)	
Race, *n* (%)			
Mexican American	408 (11.5)	132 (36.1)	< 0.001
Other hispanic	335 (9.4)	62 (16.9)	
Non‐hispanic white	1401 (39.5)	106 (29.0)	
Non‐hispanic black	828 (23.4)	24 (6.6)	
Non‐hispanic Asian	447 (12.6)	35 (9.6)	
Other race	126 (3.6)	7 (1.9)	
Education level, *n* (%)			
Less than high school	597 (16.9)	113 (30.9)	< 0.001
High school	751 (21.2)	94 (25.7)	
College	2197 (62.0)	159 (43.5)	
Marital status, *n* (%)			
Married	2155 (60.8)	223 (60.9)	0.414
Never married	437 (12.3)	39 (10.7)	
Others	953 (26.9)	104 (28.4)	
Smoking status, *n* (%)			
No	2000 (56.4)	225 (61.5)	0.170
Yes	1544 (43.6)	141 (38.5)	
Drink, *n* (%)			
No	651 (19.3)	123 (34.9)	< 0.001
Yes	2727 (80.7)	229 (65.1)	
Social economic			
Family PIR	2.61 [1.21, 4.83]	1.67 [1.00, 3.31]	< 0.001
Physical examination and activity measures			
BMI (kg/m^2^)	28.00 [24.50, 32.30]	33.55 [29.33, 39.08]	< 0.001
Weight (kg)	80.30 [68.30, 93.70]	82.15 [71.32, 100.30]	0.002
Height (cm)	168.20 [161.80, 175.20]	156.50 [150.93, 164.55]	< 0.001
AC (cm)	33.20 [30.10, 36.50]	34.80 [32.00, 38.80]	< 0.001
WC (cm)	97.30 [88.10, 107.50]	107.90 [96.90, 120.10]	< 0.001
ASM (kg)	22.71 [18.04, 27.27]	19.29 [15.91, 23.65]	< 0.001
Disease measure, *n* (%)			
Hypertension, *n* (%)			
No	2507 (70.7)	214 (58.5)	< 0.001
Yes	1037 (29.3)	152 (41.5)	
Cholesterol, *n* (%)			
No	2471 (69.7)	219 (59.8)	< 0.001
Yes	1074 (30.3)	147 (40.2)	
Diabetes, *n* (%)			
No	3184 (89.8)	285 (77.9)	< 0.001
Yes	361 (10.2)	81 (22.1)	
Other factors			
Protein (g)	79.11 [60.44, 102.36]	72.18 [54.47, 94.88]	< 0.001
Calcium (mg)	852.00 [599.50, 1170.50]	789.75 [549.38, 1073.62]	0.001
Vitamin D (μg)	3.40 [1.75, 5.95]	3.15 [1.56, 5.19]	0.040
Vitamin C (mg)	62.70 [29.45, 114.35]	53.10 [28.76, 100.96]	0.034

*Note: p* values were calculated by chi‐square test.

Abbreviations: AC, arm circumference; ASM, appendicular skeletal muscle mass; BMI, body mass index; Height, standing height; PIR, poverty‐income ratio; WC, waist circumference.

Table 2 is distinguished according to race, presenting the demographic characteristics of the study cohort. Non‐Hispanic Asians had the lowest BMI, weight, AC, WC, and ASM. Besides, family PIR, as well as retinol, alpha‐carotene, beta‐carotene, beta‐cryptoxanthin, lycopene, lutein, and zeaxanthin, were statistically significant across racial groups, indicating diverse patterns of vitamin A intake.

**TABLE 2:** Demographic characteristics by race derived from NHANES 2011–2016.

	Mexican American (*n* = 540)	Other hispanic (*n* = 397)	Non‐hispanic white (*n* = 1507)	Non‐hispanic black (*n* = 852)	Non‐hispanic Asian (*n* = 482)	*p*
Demographic						
Age, years	45 [40, 52]	48 [41, 53]	47 [41, 53]	48 [42, 53]	46 [40, 52]	< 0.0001
Sex, *n* (%)						
Male	252 (46.7)	171 (43.1)	723 (48.0)	382 (44.8)	254 (52.7)	0.044
Female	288 (53.3)	226 (56.9)	784 (52.0)	470 (55.2)	228 (47.3)	
Education, *n* (%)						
< High school	249 (44.4)	108 (27.2)	182 (12.1)	116 (13.6)	40 (8.3)	< 0.001
High school	126 (23.3)	83 (20.9)	339 (22.5)	221 (25.9)	50 (10.4)	
College	165 (30.5)	206 (51.9)	986 (65.4)	515 (60.5)	392 (81.3)	
Marital status, *n* (%)						
Married	358 (66.3)	229 (57.7)	942 (62.5)	383 (45.0)	393 (81.5)	< 0.001
Never married	40 (7.4)	43 (10.8)	154 (10.2)	193 (22.7)	32 (6.6)	
Others	142 (26.3)	125 (31.4)	411 (27.4)	276 (32.4)	57 (11.9)	
Smoking, *n* (%)						
No	340 (63.0)	236 (59.4)	704 (46.7)	528 (62.0)	357 (74.1)	< 0.001
Yes	200 (37.0)	161 (40.6)	802 (53.2)	324 (38.0)	125 (25.9)	
Drink, *n* (%)						
No	134 (26.1)	96 (25.7)	189 (12.9)	180 (22.1)	150 (34.2)	< 0.001
Yes	379 (73.9)	277 (74.3)	1273 (87.1)	636 (77.9)	288 (65.8)	
Social economic						
Family PIR	1.5 [0.9,2.9]	2.1 [1.1,3.6]	2.9 [1.2, 5.0]	2.3 [1.1, 4.1]	4.3 [2.3, 5.0]	< 0.001
Physical examination and activity measures						
BMI (kg/m^2^)	30.0 [27.0, 34.3]	29.1 [25.2, 33.2]	28.4 [24.7, 33.0]	30.1 [25.7, 35.2]	24.8 [22.6, 27.4]	< 0.001
Weight (kg)	80.2 [69.8, 92.1]	77.6 [66.3, 89.9]	83.0 [71.0, 96.5]	86.7 [73.1, 101.8]	68.2 [59.0, 77.8]	< 0.001
Height (cm)	162.0 [155.8, 169.2]	163.5 [156.6, 170.7]	170.0 [163.2, 177.1]	168.9 [163.2, 176.0]	164.1 [157.0, 171.6]	< 0.001
AC (cm)	33.8 [31.5, 36.8]	33.4 [30.1, 36.2]	33.5 [30.3, 36.6]	35.0 [31.5, 38.6]	30.3 [28.2, 32.9]	< 0.001
WC (cm)	101.0 [92.4, 109.3]	98.5 [88.8, 107.1]	99.8 [89.7, 110.2]	100.4 [89.5, 112.5]	89.0 [82.5, 96.0]	< 0.001
ASM (kg)	21.1 [17.1, 25.5]	21.0 [16.5, 25.1]	22.8 [18.1, 27.3]	24.3 [20.4, 29.9]	19.8 [15.2, 23.7]	< 0.001
Disease measure, *n* (%)						
Hypertension						
No	411 (76.1)	295 (74.3)	1089 (72.3)	460 (54.0)	373 (77.4)	< 0.001
Yes	129 (23.9)	102 (25.7)	417 (27.7)	392 (46.0)	109 (22.6)	
Cholesterol						
No	374 (69.3)	267 (67.3)	999 (66.3)	614 (72.1)	343 (71.2)	0.063
Yes	166 (30.7)	130 (32.7)	508 (33.7)	238 (27.9)	139 (28.8)	
Diabetes						
No	456 (84.4)	345 (86.9)	1382 (91.7)	729 (85.6)	438 (90.9)	< 0.001
Yes	84 (15.6)	52 (13.1)	125 (8.3)	123 (14.4)	44 (9.1)	
Vitamin A intake						
Retinol	5.8 [5.31,6.2]	5.7 [5.2, 6.1]	5.9 [5.4, 6.4]	5.6 [5.0, 6.1]	5.5 [4.9, 6.0]	< 0.001
Alpha‐carotene	4.6 [3.6, 6.0]	4.9 [3.5, 6.4]	4.2 [3.0, 6.0]	4.1 [3.0, 5.5]	5.8 [4.2, 7.0]	< 0.001
Beta‐carotene	6.9 [6.3, 7.7]	7.0 [6.2, 7.8]	7.0 [6.1, 7.8]	6.8 [5.9, 7.9]	8.0 [7.1, 8.6]	< 0.001
Beta‐cryptoxanthin	4.2 [3.3, 5.0]	4.0 [2.9, 4.8]	3.5 [2.5, 4.5]	3.7 [2.7, 4.5]	4.0 [2.9, 4.9]	< 0.001
Lycopene	8.2 [7.4, 9.0]	7.9 [6.8, 8.8]	7.9 [6.8, 8.8]	7.5 [6.1, 8.5]	7.7 [6.0, 8.7]	< 0.001
Lutein & zeaxanthin	6.8 [6.3, 7.3]	6.6 [6.1, 7.2]	6.7 [6.1, 7.4]	6.8 [6.1, 7.5]	7.3 [6.7, 8.0]	< 0.001
Other factor						
Protein (g)	81.8 [64.4,107.5]	77.0 [57.1, 100.1]	78.3 [59.7, 101.3]	75.2 [56.6, 99.0]	80.3 [62.2, 101.5]	0.001
Calcium (mg)	927.0 [690.5, 1279.5]	842.0 [581.0, 1154.5]	917.5 [637.3, 1245.0]	733.5 [522.4, 1001.0]	780.8 [552.0, 1018.8]	< 0.001
Vitamin D (μg)	3.8 [2.1, 6.2]	3.7 [2.0, 5.9]	3.4 [1.7, 5.9]	2.9 [1.5, 5.1]	3.6 [1.8, 6.4]	< 0.001
Vitamin C (mg)	68.5 [38.4, 119.6]	66.5 [34.2, 117.2]	50.3 [24.0, 96.1]	67.1 [29.4, 120.3]	79.4[44.0, 140.3]	< 0.001

*Note:* Exclusion of other races including multi‐racial. *p* values were calculated by chi‐square test.

Abbreviations: AC, arm circumference; ASM, appendicular skeletal muscle mass; BMI, body mass index; Height, standing height; PIR, poverty‐income ratio; WC, waist circumference.

### 3.2 Distribution and Correlation of Vitamin A

We analyzed retinol and six individual carotenoids used between 2011 and 2016 and applied skew correction to the log‐transformed data. Figure S2 presented the distributions of these components, revealing higher levels for lycopene, beta‐carotene, and lutein and zeaxanthin derivatives. To quantify the linear associations between them, we employed Spearman's partial correlation coefficient (Figure S3). Beta‐carotene had strong associations with alpha‐carotene (Corr. = 0.64, *p* < 0.001) and lutein and zeaxanthin (Corr. = 0.62, *p* < 0.001).

### 3.3 Exploring the Association Between VAs and Muscle Mass Levels Using GLM

Table 3 Shows the change in muscle mass associated with unit elevations in retinol and total carotenoid in men and women, respectively. For both genders, there was a marked increase observed in retinol and total carotenoid levels, while only retinol in male exhibited a significant correlation with muscle mass. It might be attributed to racial variations and the diverse composition of vitamin A components.

**TABLE 3:** Estimated associations between dietary vitamin A levels and level of muscle mass (ASM/BMI) in GLM in male and female.

	Male		Female	
	*β* ^a^ (95% CI)	*p*	*β* ^a^ (95% CI)	*p*
Retinol				
Quartile 1	Reference	—	Reference	—
Quartile 2	0.015 (−0.012, 0.042)	0.268	−0.003 (−0.023, 0.017)	0.769
Quartile 3	0.021 (−0.005, 0.047)	0.109	−0.007 (−0.025, 0.012)	0.514
Quartile 4	0.028 (0.003, 0.053)	0.027	0.018 (0.020, 0.015)	0.778
*p* for trend	—	**0.018**	—	0.438
Total carotenoid				
Quartile 1	Reference	—	Reference	—
Quartile 2	−0.010 (−0.042, 0.022)	0.543	−0.010 (−0.031, 0.012)	0.386
Quartile 3	−0.020 (−0.050, 0.010)	0.198	−0.015 (−0.035, 0.006)	0.171
Quartile 4	−0.006 (−0.035, 0.023)	0.692	−0.003 (−0.023, 0.017)	0.760
*p* for trend	—	0.188	—ssss	0.737

*Note: p*‐values for trends < 0.05 are presented in bold.

^a^ β coefficients quantified the impact of retinol and total carotenoid on the log‐odds of the outcome in GLM, adjusted for age, race, family PIR, diabetes, hypertension, and cholesterol.

### 3.4 Exploring Race‐Specific Associations Between Vitamin A Intake and Sarcopenia Using Mixture Analysis

Retinol and certain carotenoids are often concurrently consumed, and their effects may be additive, synergistic, or antagonistic. Hence, we performed two advanced mixture analysis methods to explore potential associations between sarcopenia prevalence and the intake of retinol and carotenoids. Meanwhile, we used the continuous variable ASM/BMI as the outcome. To provide a comprehensive understanding, we further stratified the analysis by gender and race, allowing us to examine these associations across diverse demographic groups.

#### 3.4.1 Vitamin A and Muscle Mass With WQS

Table 4, Illustrated that, after adjusting for all covariates, there was a significant positive correlation between total vitamin A intake and muscle mass among American adult males, whereas there was no significant positive association in women (Male: OR: 1.019, 95% CI: 1.010–1.027, *p* < 0.001; Female: OR: 1.006, 95% CI: 0.998–1.013, *p* = 0.137). In racial groups, a significant positive correlation was found for both males and females within the non‐Hispanic White and non‐Hispanic Asian populations, and specifically for women among Mexican Americans. No significant associations were found for other Hispanics and non‐Hispanic Black in either sex. Besides, the WQS regression in the negative direction did not show any significant association of the total vitamin A intake with the muscle mass in any racial group.

**TABLE 4:** The relationship between the combined effects of retinols and the six individual carotenoids and level of muscle mass (ASM/BMI) in the WQS model, differentiated by gender and race.

Male	Non‐covariates		Covariates	
	*β* (95% CI)	*p*	*β* (95% CI)	*p*
Total people	1.019 (1.009–1.029)	< 0.001	1.019 (1.010–1.027)	< 0.001
Mexican American	0.996 (0.971–1.021)	0.748	0.993 (0.970–1.017)	0.560
Other Hispanic	1.025 (0.996–1.055)	0.092	1.020 (0.993–1.047)	0.151
Non‐Hispanic White	1.033 (1.017–1.049)	< 0.001	1.028 (1.013–1.043)	< 0.001
Non‐Hispanic Black	1.015 (0.994–1.037)	0.155	1.012 (0.993–1.031)	0.213
Non‐Hispanic Asian	1.022 (1.004–1.040)	0.015	1.021 (1.003–1.039)	0.022
Female				
Total people	1.007 (1.001–1.014)	0.028	1.006 (0.998–1.013)	0.137
Mexican American	1.016 (1.002–1.030)	0.026	1.016 (1.002–1.029)	0.024
Other Hispanic	0.995 (0.975–1.015)	0.604	0.992 (0.974–1.011)	0.422
Non‐Hispanic White	1.021 (1.011–1.032)	< 0.001	1.017 (1.005–1.029)	< 0.001
Non‐Hispanic Black	1.001 (0.985–1.018)	0.867	0.995 (0.979–1.011)	0.534
Non‐Hispanic Asian	1.029 (1.012–1.047)	< 0.001	1.027 (1.010–1.045)	< 0.001

*Note:* Covariates: adjusting for age, race, family PIR, diabetes, hypertension, cholesterol, and protein intake.

The weighing analysis of vitamin A components on muscle mass was performed, and the results were observed across the sex and race subgroups, as shown in Figure S4. Table S3 Summarized the most key components with a positive impact on muscle mass, including only significant associations identified in the WQS regression. For males, higher retinol intake contributed the most to the reduced prevalence of sarcopenia, confirming the GLM results in Table 3. Lycopene intake and β‐carotene intake ranked second in contribution for non‐Hispanic White and non‐Hispanic Asian males, respectively. For females, significant racial differences were observed in the association between vitamin A intake and sarcopenia prevalence.

#### 3.4.2 Vitamin A and Muscle Mass With BKMR

**FIGURE 1:**
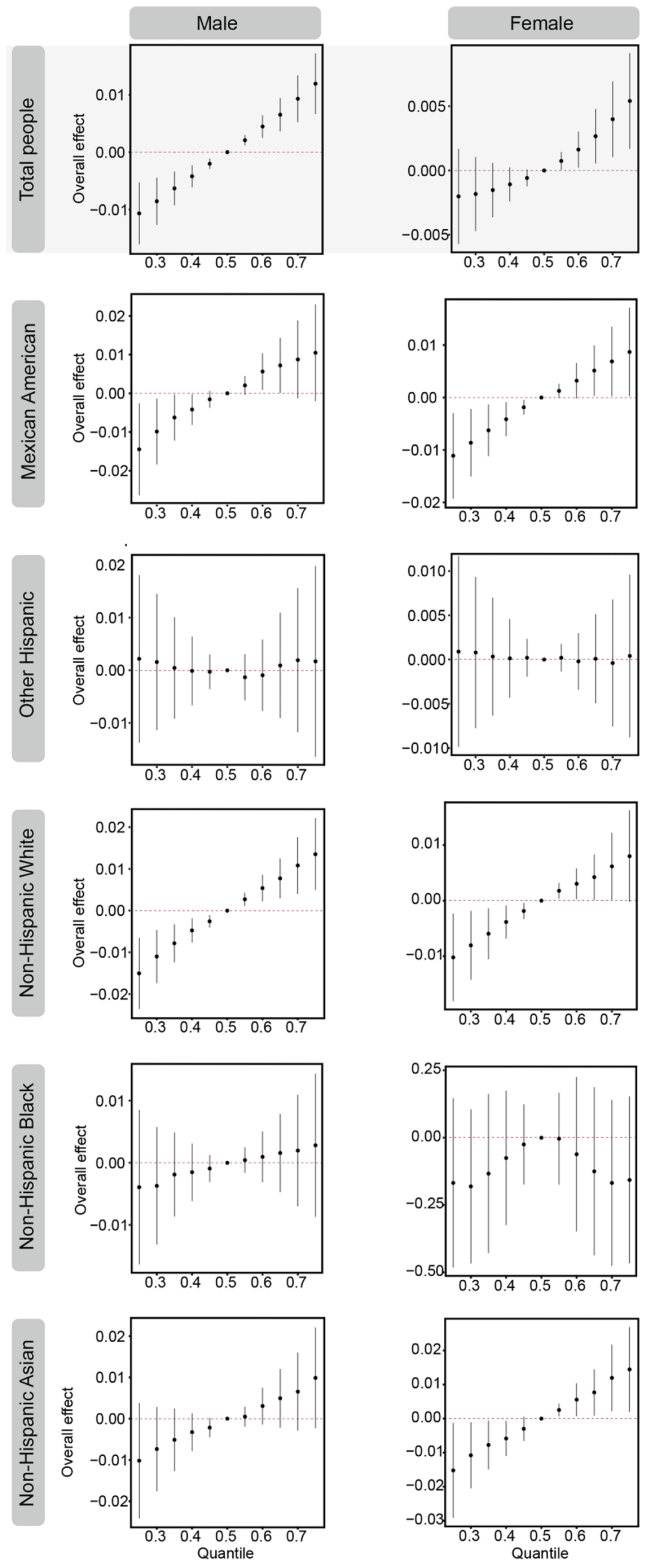
The association between total vitamin A intake and level of muscle mass (ASM/BMI) using BKMR with differentiation by sex and race. Estimates were adjusted for age (continuous), family PIR (continuous), hypertension symptoms (categorical), diabetes symptoms (categorical), cholesterol symptoms (categorical), and protein intake (continuous).

We further employed BKMR models to examine the intricate and comprehensive association between total vitamin A intake and muscle mass, accounting for differences in gender and race, as illustrated in Figure 1. Our results showed a significant positive correlation between vitamin A intake and skeletal muscle mass (ASM/BMI) in American adult males. In females, the positive association was mild at lower levels of vitamin A intake but became significant as intake increased. Among racial subgroups, a significant positive correlation was observed in both men and women of Mexican American, non‐Hispanic White, and non‐Hispanic Asian populations. It is worth noting that retinol was identified as the main driver of all males (PIP = 0.98), non‐Mexican White males (PIP = 0.42), and non‐Mexican Asian males (PIP = 0.46) influence at the 50th percentile of vitamin A intake. For females, lutein and zeaxanthin, β‐carotene, and retinol were the main drivers in Mexican Americans (PIP = 0.39), non‐Mexican White (PIP = 0.92), and non‐Mexican Asian (PIP = 0.42), respectively, as detailed in Table S4. These results were well consistent with the WQS and GLM models.

### 3.5 Mediation Analysis of Vitamin A Intake and Increased Muscle Mass

**FIGURE 2:**
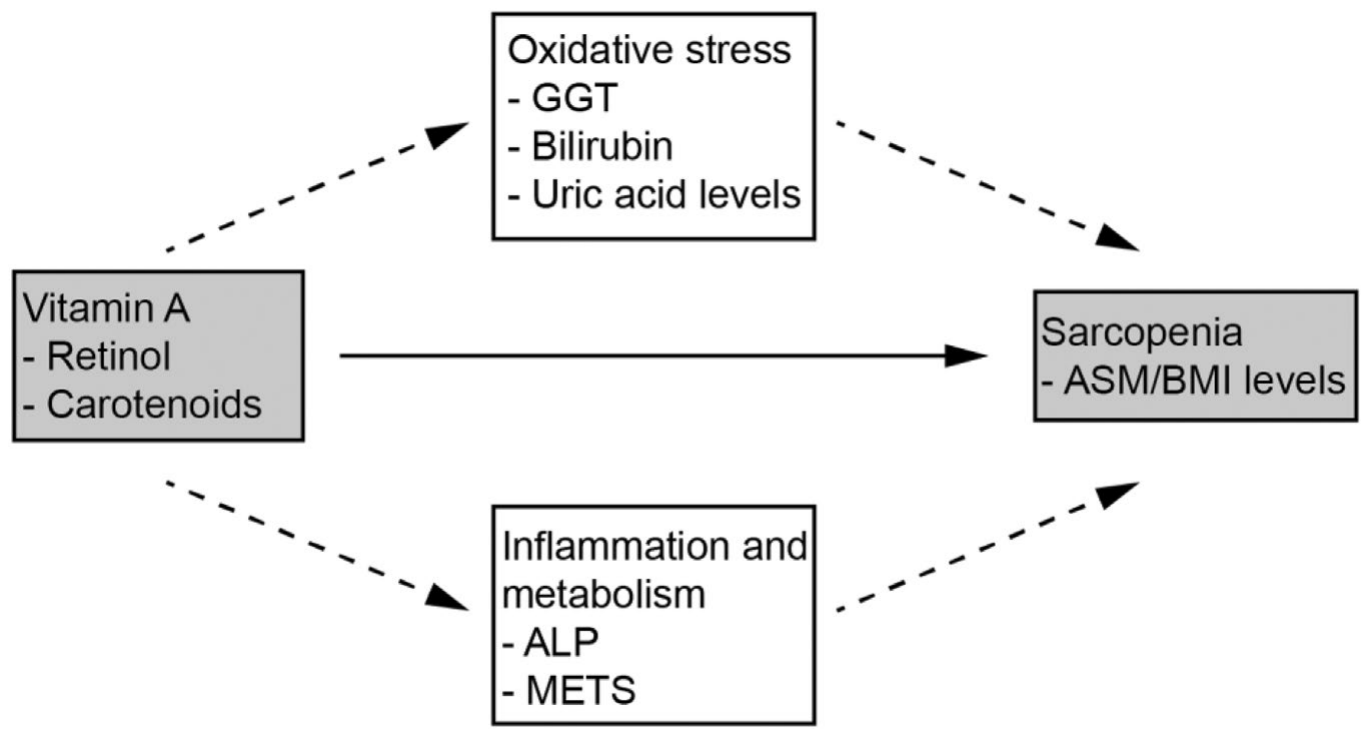
The mediating proportion of oxidative stress biomarkers quantified their role in bridging the relationship between retinol/carotenoids and ASM/BMI. Adjusted for age, race, diabetes, hypertension, and cholesterol.

We further conducted mediation analysis to explore potential causal mechanisms between vitamin A intake and the increased muscle mass in the racial populations where significant associations were identified. Figure 2 illustrates the possible pathways and the respective biomarkers, identified by the mediation analysis. As indicated in Tables S5 and S6, among American males, increased retinol intake, mediated by lowered uric acid levels, exhibited a significant correlation with higher muscle mass levels, accounting for 14.90% (*p* = 0.002) of the association. On the other hand, increased intake of carotenoids, mediated by elevated bilirubin, displayed significant associations with higher muscle mass levels, accounting for 19.90% (*p* = 0.002) of the association. Tables S7 and S8 demonstrated the relationship between retinol and carotenoids mediated by inflammation and metabolism and the level of muscle mass (ASM/BMI). Among them, there were no significant effects. The underlying biological mechanisms required further investigation through experimental studies.

## 4 Discussion

In this study, we systematically assess the gender‐ and race‐specific relationship between vitamin A intake and the sarcopenia prevalence in the 2011–2016 NHANES study population of American adults, using multiple statistical models. In the GLM model analysis, retinol was found to be significantly and negatively associated with sarcopenia in American males, while no significant association was observed in women. This may be due to racial differences and the diverse composition of VA components. We then utilized two advanced mixture analysis methods, WQS and BKMR, within the racial subgroups.

In American males, higher retinol intake was consistently associated with a reduced prevalence of sarcopenia, a result corroborated by both the GLM and mixture analysis models. Lycopene intake also showed a significant protective effect against sarcopenia in non‐Hispanic White males, while β‐carotene intake had a similar effect in non‐Hispanic Asian males. These results suggest that different vitamin A components may contribute to muscle health in a race‐specific manner, with retinol playing a central role across the male population.

For American females, the WQS model showed no significant association between vitamin A intake and muscle mass when covariates were included. Similarly, the BKMR model indicated that the positive association was mild at lower levels of vitamin A intake but became significant as intake increased. Remarkably, significant racial differences were revealed in the association between vitamin A intake and sarcopenia prevalence. Specifically, in Mexican American women, increased intake of lycopene, lutein, and zeaxanthin was significantly linked to a lower prevalence of sarcopenia. In non‐Hispanic White women, β‐carotene intake was associated with muscle mass, while in non‐Hispanic Asian women, higher retinol intake had a similar protective effect.

Mediation analysis revealed a significant association between retinol and muscle mass (ASM/BMI) in US males, with uric acid serving as a mediator. Retinol accounted for 14.90% of this relationship (*p* = 0.002), suggesting a potential mechanism linked to oxidative stress regulation. By reducing uric acid levels, retinol may help alleviate oxidative damage, thereby promoting muscle preservation and growth.

The findings of this study align with existing literature, highlighting notable sex and racial differences in muscle mass and strength. Previous research has consistently shown that men generally have higher muscle mass compared to women (Lee and Gallagher 2008; Kim et al. 2016). Furthermore, racial disparities in lean body mass, muscle strength, and physical function have been widely documented (Feng et al. 2024). For instance, the Boston Area Community Health and Bone Survey revealed that black and Hispanic men have a higher lean mass index than white men, even after adjusting for confounding factors. Similarly, the Health ABC study found that while black individuals tend to have lower muscle strength compared to their white counterparts, Asian individuals exhibit lower muscle strength relative to other racial groups (Goodpaster et al. 2006). These findings emphasize the importance of considering both sex and race when evaluating the relationship between nutrient intake and muscle health.

In terms of dietary intake, Garry reported that the median dietary retinol equivalent (RE) levels remain stable over time, at approximately 1400 RE for men and 1250 RE for women (Garry et al. 1987). The observed sex‐specific differences in the metabolism and bioavailability of vitamin A components may partially explain the differential impact on muscle mass. Experimental studies in animal models have demonstrated that the efficiency of beta‐carotene conversion to vitamin A is influenced by sex, with males exhibiting different bioavailability rates compared to females (Borel et al. 2021). This variation may help account for the observed lack of significant association between vitamin A intake and muscle mass in American women in this study, despite its protective effect in men.

Additionally, carotenoids—potent antioxidants—have been independently linked to muscle strength and physical function. Martin reported that beta‐carotene intake was associated with improved physical performance in older women, whereas no such relationship was found in men (Martin et al. 2011). This further suggests that the relationship between antioxidant nutrients, such as beta‐carotene, and muscle health may differ by sex. Another study also observed sex differences in the association between vitamin intake and hand grip strength, a key marker of sarcopenia, with disparities in the role of vitamins A, C, and E, as well as carotenoids and minerals like selenium and zinc (Wu et al. 2023).

In our study, we found that retinol is the main reason why vitamin A affects sarcopenia. A research study demonstrated that elevated levels of circulating retinol positively correlate with muscle mass and strength among school‐aged girls (Lecoq, Chauchard, and Mazoue 1946), which is consistent with our results. And mechanistically, vitamin A exerts differential effects on the osteogenic phase by facilitating early osteoblast differentiation while suppressing bone mineralization, mediated by retinoic acid receptor signaling and the regulation of osteoblast/osteoclast‐associated bone peptides. However, adequate intake of vitamin A through food or supplements has been shown to maintain bone health. Also, pro‐vitamin A (carotenoids and beta‐cryptoxanthin) protects bones.

As the reasons for how vitamin A affects muscle mass are not unique, we mediated analyses of oxidative stress, inflammation, and metabolism. Depletion of antioxidant nutrients and fatty acids can help maintain muscle mass by reducing oxidative stress, and vitamin A and carotenoids, often considered members of the antioxidant vitamin family, can exhibit beneficial effects in mitigating oxidative stress and certain chronic ailments. While aerobic metabolism boasts numerous benefits, it is partially counterbalanced by potentially harmful physiological impacts arising from highly reactive oxygen species and their reduction products. In response, nature has evolved diverse and intricate defense mechanisms to mitigate these unfavorable oxygen‐related side effects (Olson 1996). For oxidative stress analysis, vitamin A, through its structure and potential free radical quenching effects, apparently induces more oxidative stress in skeletal muscle, leading to tissue damage. For oxidatively modified proteins, the ET group had a significant reduction in total thiol content, which may indicate elevated glutathione disulfide (GSSG) levels. GSSG is often used as a marker of a system's response to oxidative stress, as its detection shows that the GSH group is actively involved in redox reactions (Magalhães et al. 2007). In addition, for inflammation and metabolism, the association between inflammation and vitamin A metabolism and state assessment has been demonstrated in several animal and human studies, and dietary carotenoids and retinoids have been documented to play an important role in innate and acquired immunity and the body's response to inflammation (Rubin et al. 2017).

This study utilized diverse statistical techniques and enabled a comprehensive evaluation of the association between vitamin A intake and sarcopenia. GLM was employed to contrast the risk of vitamin A exposure before and after adjusting for various covariates. In the WQS regression, exposure values were quantized and amalgamated into a single weighted index, facilitating dimensionality reduction and circumventing multicollinearity. The concurrent deployment of these methods would facilitate the estimation of the aggregate effect of each exposure and its impact amidst potential interactions. Furthermore, the utilization of the BKMR model further mitigated the confounding influence of interacting factors.

There are also some limitations in our study that are worth noting. First, cross‐sectional analyses inherently constrain our ability to draw causal inferences, and potential selection bias remains a concern. Second, while we analyzed data from three NHANES periods, additional prospective studies are required to further corroborate our findings. Third, the limitations inherent in self‐reported dietary data, including potential recall bias and measurement error, might affect the accuracy of vitamin A intake assessments and the overall findings. Fourthly, we used the continuous variable ASM/BMI as the indicator of sarcopenia. While it is a key metric, it might somewhat lack clinical significance. Notably, the BKMR model excluded weighting factors due to the absence of corresponding arguments, potentially limiting the generalizability of its results. Lastly, unmeasured confounders may introduce residual bias into our findings.

## 5 Conclusion

Our study identified an overall negative correlation between vitamin A intake and sarcopenia prevalence. Specifically, retinol predominantly influenced males, while certain carotenoids had a significant impact on females. Furthermore, oxidative stress was found to mediate the relationship between vitamin A intake and sarcopenia prevalence in both genders.

## Author Contributions

**Meisi Fang:** formal analysis (equal), methodology (equal), writing – original draft (equal). **Jinghang Ma:** writing – original draft (equal). **Yongyu Bai:** resources (equal), writing – original draft (equal). **Yifan Ying:** conceptualization (equal), resources (equal), writing – original draft (equal). **Xian Shen:** project administration (equal), writing – review and editing (equal).

## Ethics Statement

We were not involved in recruiting the participants because this paper relied on publicly accessible data from U.S. NHANES. Because the written informed consent was obtained from all participants as part of the NHANES process, this study was exempt from requiring ethics review approval.

## Consent

The authors have nothing to report.

## Conflicts of Interest

The authors declare no conflicts of interest.

## Supporting information


Appendix S1.

## Data Availability

The datasets generated and/or evaluated as part of this research are available in the NHANES repository (https://www.cdc.gov/nchs/nhanes/index.htm).
