# Supplementary material for: Systematic Exploration of the Association Between Vitamin A Intakes and Sarcopenia Prevalence in American Adults
Source: Food Sci Nutr. 2024 Nov 25;12(12):10786–99. doi: 10.1002/fsn3.4613 (PMC11666842; doi:10.1002/fsn3.4613)
Supplement: Supplementary file 1 — Appendix S1. [file FSN3-12-10786-s001.docx]

**Supplemental Materials**

**Supplementary Tables**

**Table S1** Demographic Characteristics by Race in the Male Population of the NHANES Study

|  | **Mexican American**  **(n=252)** | **Other Hispanic**  **(n=171)** | **Non-Hispanic White**  **(n=723)** | **Non-Hispanic Black**  **(n=382)** | **Non-Hispanic Asian**  **(n=254)** | **P-value** |
| --- | --- | --- | --- | --- | --- | --- |
| **Demographic** | | | | | | |
| Age, years | 45[39, 52] | 47 [40, 52] | 47[41, 53] | 47[42, 54] | 46[41, 52] | 0.02 |
| Education, n (%) |  |  |  |  |  | <.001 |
| <High school | 121 (48.0) | 50 (29.3) | 96 (13.3) | 62 (16.3) | 25 (9.9) |  |
| High school | 63 (25.0) | 38 (22.2) | 175 (24.2) | 111 (29.1) | 25 (9.8) |  |
| College | 68 (26.9) | 83 (48.6) | 452 (62.5) | 209 (54.7) | 204 (80.3) |  |
| Marital status, n (%) |  |  |  |  |  | <.001 |
| Married | 175 (69.4) | 110 (64.3) | 470 (65.0) | 204 (53.4) | 217 (85.4) |  |
| Never married | 18 (7.1) | 16 (9.4) | 89 (12.3) | 75 (19.6) | 14 (5.5) |  |
| Others | 59(23.4) | 45 (26.3) | 164 (22.8) | 103 (26.9) | 23 (9.0) |  |
| Smoking, n (%) |  |  |  |  |  | <.001 |
| No | 123 (48.8) | 80 (46.8) | 327 (45.2) | 207 (54.2) | 160 (63.0) |  |
| Yes | 129 (51.2) | 91 (53.2) | 395 (54.6) | 175 (45.8) | 94 (37.0) |  |
| Drink, n (%) |  |  |  |  |  | <.001 |
| No | 25 (10.4) | 22 (13.4) | 57 (8.0) | 52 (14.1) | 52 (21.8) |  |
| Yes | 216 (89.6) | 142 (86.6) | 654 (92.0) | 316 (85.9) | 186 (78.2) |  |
| **Social economic** | | | | | | |
| Family PIR | 1.5[1.0, 2.9] | 2.2[1.1, 3.5] | 3.0 [1.2, 5.0] | 2.5[1.2, 4.3] | 4.4[2.5, 5.0] | <.001 |
| Physical examination and activity measures | | | | | | |
| BMI (kg/$m^{2}$) | 29.3[26.7, 32.5] | 28.6[26.0, 31.5] | 28.4[25.5, 32.1] | 28.7[24.7, 33.2] | 25.3[23.5, 27.5] | <.001 |
| Weight (kg) | 83.9[75.6, 94.4] | 83.8[74.7, 94.0] | 89.3[78.7, 101.8] | 89.7[76.6, 104.6] | 72.9[67.2, 81.7] | <.001 |
| Height (cm) | 169.2[164.7, 173.4] | 171.1[166.9, 175.8] | 176.9[172.7, 181.2] | 176.8[172.0, 181.2] | 170.9[165.8, 174.8] | <.001 |
| AC (cm) | 34.2[32.2, 36.7] | 34.4[32.0, 36.9] | 34.7[32.0, 37.2] | 35.7[32.2, 39.4] | 31.4[29.7, 33.6] | <.001 |
| WC (cm) | 101.2[93.3, 108.7] | 100.5[92.0 108.1] | 103.1[94.4, 111.2] | 100.2[88.5, 111.5] | 92.3[86.2, 96.8] | <.001 |
| ASM (kg) | 25.4[23.1, 28.4] | 25.5[23.3, 29.0] | 27.3[24.4, 30.2] | 30.1[26.3, 34.1] | 23.5[21.2, 25.6] | <.001 |
| **Disease measure, n (%)** | | | | | | |
| Hypertension |  |  |  |  |  | <.001 |
| No | 197 (78.2) | 127 (74.3) | 498 (68.9) | 203 (53.1) | 192 (75.6) |  |
| Yes | 55 (21.8) | 44 (25.7) | 225 (31.1) | 179 (46.9) | 62 (24.4) |  |
| Cholesterol |  |  |  |  |  | .071 |
| No | 162 (64.3) | 121 (70.8) | 452 (62.5) | 271 (70.9) | 168 (66.1) |  |
| Yes | 90 (35.7) | 50 (29.2) | 271 (37.5) | 111 (29.1) | 86 (33.9) |  |
| Diabetes |  |  |  |  |  | .004 |
| No | 216 (85.7) | 149 (87.1) | 662 (91.6) | 321 (84.0) | 229 (90.2) |  |
| Yes | 36 (14.3) | 22 (12.9) | 61 (8.4) | 61 (16.0) | 25 (9.8) |  |
| **Vitamin A intake** | | | | | | |
| Retinol | 5.8[5.4, 6.3] | 5.7[5.3, 6.3] | 6.0 [5.4, 6.4] | 5.8[5.1, 6.2] | 5.5[4.9, 6.0] | <.001 |
| Alpha-carotene | 4.3[3.6, 5.9] | 4.6[3.5, 6.3] | 4.2 [3.1, 6.0] | 4.1[2.8, 5.6] | 5.9[4.1, 7.1] | <.001 |
| Beta-carotene | 6.9 [6.2, 7.6] | 7.0[6.2, 7.7] | 6.9 [6.1, 7.8] | 6.8[5.8, 7.9] | 8.0[7.1, 8.6] | <.001 |
| Beta-cryptoxanthin | 4.3 [3.5, 5.0] | 4.0[3.0, 4.8] | 3.5 [2.6, 4.5] | 3.8[2.7, 4.6] | 4.0[2.9, 4.7] | <.001 |
| Lycopene | 8.5 [7.5, 9.2] | 8.0[7.2, 8.8] | 8.0 [6.9, 8.9] | 7.8[6.2, 8.9] | 7.7[5.8, 8.7] | <.001 |
| Lutein & zeaxanthin | 6.8 [6.3, 7.3] | 6.7[6.1, 7.1] | 6.8 [6.1, 7.4] | 6.8[6.2, 7.5] | 7.3[6.7, 8.0] | <.001 |
| **Other facto** | | | | | | |
| Protein (g) | 98.3[74.7, 126.2] | 87.8[65.0, 113.2] | 92.5[71.7, 117.1] | 89.7[67.3, 116.7] | 90.4[71.2, 117.8] | .025 |
| Calcium (mg) | 1070.3 [770.0, 1489.6] | 920.5[667.8, 1291.0] | 1020.5[711.3, 1382.3] | 814.0[548.0, 1100.0] | 836.8[612.1, 1057.5] | <.001 |
| Vitamin D (μg) | 3.9 [2.5, 7.2] | 4.0[2.3, 7.0] | 4.0[2.0, 7.3] | 3.5[1.8, 6.1] | 4.10[2.0, 7.0] | .019 |
| Vitamin C (mg) | 68.9[37.0, 126.1] | 68.8[31.4, 114.9] | 49.3[24.5, 105.6] | 68.6[30.2, 127.5] | 83.2[46.7, 151.0] | <.001 |

**Table S2** Demographic Characteristics by Race in the Female Population of the NHANES Study

|  | **Mexican American**  **(n=288)** | **Other Hispanic**  **(n=226)** | **Non-Hispanic White**  **(n=784)** | **Non-Hispanic Black**  **(n=470)** | **Non-Hispanic Asian**  **(n=228)** | **P-value** |
| --- | --- | --- | --- | --- | --- | --- |
| **Demographic** | | | | | | |
| Age, years | 45.[40, 51] | 48[42, 53] | 47[42, 53] | 48[42,53] | 46[40,52] | <.001 |
| Education, n (%) |  |  |  |  |  | <.001 |
| <High school | 128 (44.5) | 58 (25.6) | 86 (11.0) | 54 (11.5) | 15 (6.6) |  |
| High school | 63 (21.9) | 45 (19.9) | 164 (20.9) | 110 (23.4) | 25 (11.0) |  |
| College | 97 (33.7) | 123 (54.4) | 534 (68.1) | 306 (65.1) | 188 (82.4) |  |
| Marital status, n (%) |  |  |  |  |  | <.001 |
| Married | 183 (63.5) | 119 (52.7) | 472 (60.2) | 179 (38.1) | 176 (77.2) |  |
| Never married | 22 (7.6) | 27 (11.9) | 65 (8.3) | 118 (25.1) | 18 (7.9) |  |
| Others | 83 (28.8) | 80 (35.3) | 247 (31.5) | 173 (36.8) | 34 (14.9) |  |
| Smoking, n (%) |  |  |  |  |  | <.001 |
| No | 217 (75.3) | 156 (69.0) | 377 (48.1) | 321 (68.3) | 197 (86.4) |  |
| Yes | 71 (24.7) | 70 (31.0) | 407 (51.9) | 149 (31.7) | 31 (13.6) |  |
| Drink, n (%) |  |  |  |  |  | <.001 |
| No | 109 (40.1) | 74 (35.4) | 132 (17.6) | 128 (28.6) | 98 (49.0) |  |
| Yes | 163 (59.9) | 135 (64.6) | 619 (82.4) | 320 (71.4) | 102 (51.0) |  |
| **Social economic** | | | | | | |
| Family PIR | 1.5[0.9,2.9] | 2.1[1.1,3.7] | 2.8[1.3, 5.0] | 2.2[1.1, 3.9] | 3.9[2.0, 5.0] | <.001 |
| Physical examination and activity measures | | | | | | |
| BMI (kg/$m^{2}$) | 31.2[27.2, 36.1] | 30.0[23.8, 33.8] | 28.5[23.7, 33.8] | 31.3[26.6, 36.4] | 24.1[21.6, 27.3] | <.001 |
| Weight (kg) | 76.5[65.6, 89.5] | 73.9[61.3, 85.4] | 75.9[65.0, 90.4] | 84.3[70.5, 97.6] | 59.4[53.7, 68.8] | <.001 |
| Height (cm) | 156.7[152.1, 160.6] | 158.0[153.5, 162.8] | 163.7[159.7, 168.1] | 164.1 [160.1, 167.6] | 157.2 [154.0, 162.2] | <.001 |
| AC (cm) | 33.2[30.4, 37.1] | 32.3[28.6, 35.5] | 32.0[28.6, 36.0] | 34.5[30.8, 37.9] | 28.7[26.5, 31.2] | <.001 |
| WC (cm) | 100.4[91.7, 110.1] | 96.2[85.9, 106.2] | 96.6[85.8, 108.6] | 100.8[90.0, 113.8] | 85.1[80.0, 92.7] | <.001 |
| ASM (kg) | 76.5[65.6, 89.5] | 73.9[61.3, 85.4] | 75.9[65.0, 90.4] | 84.3[70.5, 97.6] | 59.4[53.7, 68.8] | <.001 |
| **Disease measure, n (%)** | | | | | | |
| Hypertension |  |  |  |  |  | <.001 |
| No | 214 (74.3) | 168 (74.3) | 591 (75.5) | 257 (54.7) | 181 (79.4) |  |
| Yes | 74 (25.7) | 58 (25.7) | 192 (24.5) | 213 (45.3) | 47 (20.6) |  |
| Cholesterol |  |  |  |  |  | .047 |
| No | 212 (73.6) | 146 (64.6) | 547 (69.8) | 343 (73.0) | 175 (76.8) |  |
| Yes | 76 (26.4) | 80 (35.4) | 237 (30.2) | 127 (27.0) | 53 (23.2) |  |
| Diabetes |  |  |  |  |  | .001 |
| No | 240 (83.3) | 196 (86.7) | 720 (91.8) | 408 (86.8) | 209 (91.7) |  |
| Yes | 48 (16.7) | 30 (13.3) | 64 (8.2) | 62 (13.2) | 19 (8.3) |  |
| **Vitamin A intake** | | | | | | |
| Retinol | 5.7[5.3, 6.1] | 5.6[5.1, 6.1] | 5.8[5.3, 6.2] | 5.5[5.0, 6.0] | 5.4[4.9, 6.0] | <.001 |
| Alpha-carotene | 4.8[3.7, 6.2] | 5.0[3.5, 6.4] | 4.2[3.0, 6.0] | 4.1[3.1, 5.5] | 5.8[4.3, 6.9] | <.001 |
| Beta-carotene | 7.0[6.3, 7.8] | 7.0[6.2, 7.8] | 7.0[6.0, 7.9] | 6.9[5.9, 8.0] | 8.0[7.0, 8.6] | <.001 |
| Beta-cryptoxanthin | 4.1[3.2, 4.9] | 4.0[2.9, 4.8] | 3.5[2.4, 4.4] | 3.7[2.7, 4.4] | 4.1[3.0, 5.0] | <.001 |
| Lycopene | 8.1[7.2, 8.9] | 8.0[6.7, 8.7] | 7.8[6.6, 8.7] | 7.4[6.1, 8.3] | 7.6[6.2, 8.7] | <.001 |
| Lutein & zeaxanthin | 6.8[6.3, 7.3] | 6.6[6.1, 7.2] | 6.7 [6.1, 7.4] | 6.7[6.1, 7.5] | 7.3[6.8, 8.0] | <.001 |
| **Other factor** | | | | | | |
| Protein (g) | 71.9[57.8, 90.7] | 69.3[53.49, 92.7] | 68.3[51.4, 85.1] | 65.9[52.0, 84.4] | 72.1[55.6, 87.7] | .014 |
| Calcium (mg) | 847.0[630.4, 1139.6] | 781.5[558.3, 1014.8] | 833.0[581.0, 1118.6] | 692.3[510.0, 920.3] | 720.8[509.4, 950.0] | <.001 |
| Vitamin D (μg) | 3.4[1.8, 5.5] | 3.5[1.9, 5.4] | 3.0[1.5, 5.1] | 2.5[1.4, 4.4] | 3.1[1.8, 5.3] | <.001 |
| Vitamin C (mg) | 66.4[39.3, 115.8] | 66.1[35.9, 119.2] | 51.3[23.1, 92.6] | 64.6[29.2, 110.4] | 77.4[42.8, 126.2] | <.001 |

**Table S3** Summary of positively weighted values for retinol and individual carotenoids in relation to muscle mass (ASM/BMI) as estimated by the WQS model.

|  |  | **Retinol** | | **α-carotene** | | **β-carotene** | | **β-cryptoxanthin** | | **lycopene** | | **lutein& zeaxanthin** | |
| --- | --- | --- | --- | --- | --- | --- | --- | --- | --- | --- | --- | --- | --- |
| **Male** | Total | 1 | 1 |  |  |  |  |  |  |  | 2 |  |  |
|  | Non-Hispanic White | 1 | 1 |  |  |  |  |  |  | 2 | 2 |  |  |
|  | Non-Hispanic Asian | 1 | 1 |  |  | 2 | 2 |  |  |  |  |  |  |
| **Female** | Mexican American |  |  |  |  |  |  |  | 3 | 1 | 1 | 2 | 2 |
|  | Non-Hispanic White |  | 1 |  |  | 1 | 2 |  |  |  |  |  |  |
|  | Non-Hispanic Asian | 1 | 1 |  |  |  | 2 |  |  |  |  | 2 |  |

Note: Only significant associations were included. The numbers represented the rank of each exposure in the WQS regression. Left and right panel showed the results without and with covariates, respectively.

**Table S4** Posterior inclusion probability (PIP) estimation for each Vitamin A exposure in relation to muscle mass (ASM/BMI) using the BKMR method

| **Male** | | | | | | |
| --- | --- | --- | --- | --- | --- | --- |
| **Vitamin A subclass** | Total people | Mexican American | Other Hispanic | Non-Hispanic White | Non-Hispanic Black | Non-Hispanic Asian |
| Retinol | **0.98** | 0.10 | 0.03 | **0.42** | 0.02 | **0.46** |
| Alpha-carotene | 0 | 0 | 0.00 | 0 | 0 | 0.00 |
| Beta-carotene | 0 | 0.11 | 0.03 | 0.03 | 0.01 | **0.00** |
| Beta-cryptoxanthin | 0 | 0.00 | 0.06 | **0.11** | 0.00 | 0.00 |
| Lycopene | 0 | 0.00 | 0.04 | 0 | 0 | 0 |
| Lutein & zeaxanthin | 0.01 | 0.05 | 0.03 | 0.42 | 0.02 | **0.01** |
| **Female** | | | | | | |
| **Vitamin A subclass** | Total people | Mexican American | Other Hispanic | Non-Hispanic White | Non-Hispanic Black | Non-Hispanic Asian |
| Retinol | 0.00 | **0.01** | 0.02 | 0.82 | 0.09 | **0.42** |
| Alpha-carotene | 0 | 0.01 | 0.00 | 0.77 | 0.07 | 0.01 |
| Beta-carotene | 0 | **0.01** | 0.01 | **0.92** | 0.06 | 0.06 |
| Beta-cryptoxanthin | 0 | 0 | 0.00 | 0.81 | 0.07 | 0.00 |
| Lycopene | 0 | 0.00 | 0.00 | 0.84 | 0.07 | 0 |
| Lutein & zeaxanthin | 0.51 | **0.39** | 0.06 | 0.84 | 0.10 | **0.32** |

Note: Significant results were represented in bold.

**Table S5** Mediating effect and proportions of oxidative stress biomarkers between retinol and carotenoids exposure and the prevalence of muscle mass (ASM/BMI) in male.

| Race | Pathways | Direct effect | 95%CI | Mediation proportions | 95%CI | P-value |
| --- | --- | --- | --- | --- | --- | --- |
| Total people | retinol->GGT->Sarcopenia  (-2.46) | 7.41e-03 | 1.11e-03,0.01 | 3.09% | -5.95e-03,0.18 | .090 |
|  | retinol->Bilirubin->Sarcopenia  (0.01) | 7.42e-03 | 4.94e-04,0.01 | 2.95% | -2.63e-02,0.18 | .148 |
|  | **retinol->Uric acid->Sarcopenia**  **(-0.14)** | **6.50e-03** | **6.68e-05,0.01** | **14.90%** | **3.24e-02,0.63** | **.002** |
|  | carotenoids->GGT->Sarcopenia  (-1.34) | 1.53e-03 | -3.13e-03,0.01 | 8.06% | -6.89e-01,0.82 | .140 |
|  | carotenoids->Bilirubin->Sarcopenia  (0.01) | 1.41e-03 | -3.03e-03,0.01 | 15.70% | -1.53e+00,1.76 | .064 |
|  | carotenoids->Uric acid->Sarcopenia  (-0.07) | 1.07e-03 | -3.34e-03,0.01 | 35.50% | -2.86e+00,2.44 | .012 |
| Non-  Hispanic White | retinol->GGT->Sarcopenia  (-3.27) | 9.36e-03 | -2.38e-03,0.02 | 6.66% | -0.18,0.59 | .080 |
|  | retinol->Bilirubin->Sarcopenia  (0.00) | 9.96e-03 | -2.04e-03,0.02 | 0.68% | -0.16,0.17 | .860 |
|  | retinol->Uric acid->Sarcopenia  (-0.16) | 8.49e-03 | -2.83e-03,0.00 | 15.35% | -0.66,0.94 | .022 |
|  | carotenoids->GGT->Sarcopenia  (0.89) | 2.19e-03 | -5.32e-03,0.01 | -9.53% | -0.93,1.07 | .440 |
|  | carotenoids->Bilirubin->Sarcopenia  (0.02) | 1.66e-03 | -6.18e-03,0.01 | 17.14% | -1.12,1.12 | .250 |
|  | carotenoids->Uric acid->Sarcopenia  (-0.02) | 1.83e-03 | -6.23e-03,0.01 | 8.61% | -1.36,1.10 | .760 |
| Non-  Hispanic Asian | retinol->GGT->Sarcopenia  (-3.34) | 1.11e-02 | -2.62e-03,0.03 | 5.18% | -0.27,0.43 | .400 |
|  | retinol->Bilirubin->Sarcopenia  (0.03) | 1.07e-02 | -3.28e-03,0.02 | 7.92% | -0.34,0.88 | .200 |
|  | retinol->Uric acid->Sarcopenia  (-0.21) | 1.24e-02 | -2.54e-03,0.03 | -6.08% | -0.95,0.61 | .526 |
|  | carotenoids->GGT->Sarcopenia  (0.89) | -9.50e-03 | -2.25e-02,0.00 | -8.38% | -0.82,0.77 | .110 |
|  | carotenoids->Bilirubin->Sarcopenia  (-0.02) | -8.22e-03 | -1.96e-02,0.00 | 6.25% | -0.54,0.81 | .380 |
|  | carotenoids->Uric acid->Sarcopenia  (-0.09) | -8.63e-03 | -2.04e-02,0.00 | 1.60% | -0.51,0.40 | .830 |

**Table S6** Mediating effect and proportions of oxidative stress biomarkers between retinol and carotenoids exposure and the prevalence of muscle mass (ASM/BMI) in female.

|  | Pathways | Direct effect | 95%CI | Mediation proportions | 95%CI | P-value |
| --- | --- | --- | --- | --- | --- | --- |
| Total people | retinol->GGT->Sarcopenia  (-1.87) | -1.04e-03 | -6.20e-03,0.00 | -7.74% | -0.94,0.57 | .600 |
|  | retinol->Bilirubin->Sarcopenia  (-0.01) | -4.05e-04 | 5.43e-03,0.00 | 55.82% | -2.51,4.14 | .090 |
|  | retinol->Uric acid->Sarcopenia  (-0.14) | -2.62e-03 | -7.72e-03,0.00 | -172.23% | -9.09,9.43 | <2e-16 |
|  | carotenoids->GGT->Sarcopenia  (-1.87) | 1.10e-06 | 7.75e-07,0.00 | 0.40% | -1.30e-02,0.02 | .168 |
|  | carotenoids ->Bilirubin->Sarcopenia  (0.02) | 3.06e-03 | -9.69e-04,0.01 | 18.78% | -0.74,1.13 | <2e-16 |
|  | carotenoids->Uric acid->Sarcopenia  (-0.04) | 3.33e-03 | -5.06e-04,0.01 | -11.60% | -0.13,0.73 | .144 |
| Mexican American | retinol->GGT->Sarcopenia  (-4.26) | -7.20e-03 | -1.74e-02,0.00 | -1.25% | -0.54,0.58 | .980 |
|  | retinol->Bilirubin->Sarcopenia  (0.00) | -7.23e-03 | -1.88e-02,0.00 | -1.65% | -0.78,0.66 | .840 |
|  | retinol->Uric acid->Sarcopenia  (-0.01) | -7.14e-03 | -1.78e-02,0.00 | -0.32% | -0.40,0.22 | .950 |
|  | carotenoids->GGT->Sarcopenia  (-16.47) | 8.98e-03 | -4.00e-04,0.02 | 1.83% | -0.61,0.60 | .826 |
|  | carotenoids->Bilirubin->Sarcopenia  (0.03) | 8.26e-03 | -7.97e-04,0.02 | 9.73% | -0.13,0.64 | .262 |
|  | carotenoids->Uric acid->Sarcopenia  (0.17) | 9.76e-03 | 5.43e-04,0.02 | -6.60% | -0.54,0.16 | .256 |
| Non-Hispanic White | retinol->GGT->Sarcopenia  (-4.67) | -9.58e-04 | -1.09e-02,0.01 | -6.75% | -1.57,0.91 | .510 |
|  | retinol->Bilirubin->Sarcopenia  (-0.03) | 6.79e-04 | -9.74e-03,0.01 | 218.77% | -3.04,3.68 | .048 |
|  | retinol->Uric acid->Sarcopenia  (-0.14) | -2.99e-03 | -1.29e-02,0.01 | -426.99% | -5.81,6.68 | .022 |
|  | carotenoids->GGT->Sarcopenia  (-1.28) | 8.49e-03 | 2.07e-03,0.02 | 1.15% | -6.05e-02,0.14 | .610 |
|  | **carotenoids->Bilirubin->Sarcopenia**  **(0.04)** | **6.88e-03** | **6.82e-04,0.01** | **19.90%** | **5.41e-02,0.68** | **.002** |
|  | carotenoids->Uric acid->Sarcopenia  (-0.03) | 8.11e-03 | 1.09e-03,0.01 | 5.59% | -0.22,0.41 | .482 |
| Non-Hispanic Asian | retinol->GGT->Sarcopenia  (5.49) | 1.54e-02 | 2.73e-03,0.03 | -7.97% | -0.37,0.07 | .196 |
|  | retinol->Bilirubin->Sarcopenia  (-0.02) | 1.53e-02 | 3.47e-03,0.03 | -7.13% | -0.53,0.12 | .352 |
|  | retinol->Uric acid->Sarcopenia  (-0.27) | 1.25e-02 | -6.85e-05,0.03 | 12.50% | -4.39e-02,0.75 | .106 |
|  | carotenoids->GGT->Sarcopenia  (4.74) | 4.28e-03 | -6.88e-03,0.01 | -22.53% | -2.08,1.23 | .500 |
|  | carotenoids ->Bilirubin->Sarcopenia  (-0.04) | 5.59e-03 | -4.84e-03,0.02 | -5.99% | -5.41,8.02 | .460 |
|  | carotenoids->Uric acid->Sarcopenia  (-0.19) | 1.81e-03 | -8.94e-03,0.01 | 48.30% | -3.93e,5.06 | .066 |

**Table S7** Mediating effect and proportions of inflammation and metabolism between retinol and carotenoids exposure and the prevalence of muscle mass (ASM/BMI) in male.

|  | Pathways | Indirect effect | 95%CI | Mediation proportions | 95%CI | P-value |
| --- | --- | --- | --- | --- | --- | --- |
| Total people | Inflammation |  |  |  |  |  |
|  | retinol->ALP->Sarcopenia  (0.83) | 8.02e-03 | 1.84e-03,0.01 | -4.95% | -0.32,0.02 | .120 |
|  | carotenoids->ALP->Sarcopenia  (-0.58) | 1.41e-03 | -3.27e-03,0.01 | 15.57% | -1.29,1.27 | .260 |
|  | Metabolism |  |  |  |  |  |
|  | retinol->METS->Sarcopenia  (0.67) | 7.77e-03 | 1.22e-03,0.01 | -1.73% | -0.12,0.07 | .508 |
|  | carotenoids->METS->Sarcopenia  (0.12) | 1.69e-03 | -3.32e-03,0.01 | -1.33% | -0.38,0.47 | .880 |
| Non-  Hispanic White | Inflammation |  |  |  |  |  |
|  | retinol->ALP->Sarcopenia  (1.93) | 1.04e-02 | -1.00e-03,0.02 | -4.02% | -0.37,0.20 | .232 |
|  | carotenoids->ALP->Sarcopenia  (-1.15) | 1.79e-03 | -5.82e-03,0.01 | 10.41% | -0.70,1.31 | .500 |
|  | Metabolism |  |  |  |  |  |
|  | retinol->METS->Sarcopenia  (0.87) | 1.05e-02 | -1.23e-03,0.02 | -4.83% | -0.47,0.37 | .306 |
|  | carotenoids->METS->Sarcopenia  (0.24) | 2.13e-03 | -5.76e-03,0.01 | -6.53% | -0.98,0.94 | .620 |
| Non-  Hispanic Asian | Inflammation |  |  |  |  |  |
|  | retinol->ALP->Sarcopenia  (-0.17) | 1.16e-02 | -3.13e-03,0.03 | -1.05% | -0.39,0.44 | .990 |
|  | carotenoids->ALP->Sarcopenia  (-2.08) | -1.05e-02 | -2.20e-02,0.00 | -20.00% | -1.89,1.26 | .080 |
|  | Metabolism |  |  |  |  |  |
|  | retinol->METS->Sarcopenia  (-0.58) | 1.11e-02 | -2.92e-03,0.03 | -4.45% | -0.55,0.45 | .640 |
|  | carotenoids->METS->Sarcopenia  (-0.03) | -8.79e-03 | -2.00e-02,0.00 | -0.28% | -0.52,0.58 | .990 |

**Table S8** Mediating effect and proportions of inflammation and metabolism between retinol and carotenoids exposure and the prevalence of muscle mass (ASM/BMI) in female.

|  | Pathways | Indirect effect | 95%CI | Mediation proportions | 95%CI | P-value |
| --- | --- | --- | --- | --- | --- | --- |
| Total people | Inflammation |  |  |  |  |  |
|  | retinol->ALP->Sarcopenia  (-0.51) | 1.17e-03 | 5.97e-03,0.00 | -2.11% | -1.69,1.34 | .460 |
|  | carotenoids->ALP->Sarcopenia  ( -2.15) | 2.92e-03 | -9.07e-04,0.01 | 2.24% | -0.31,1.50 | <2e-16 |
|  | Metabolism |  |  |  |  |  |
|  | retinol->METS->Sarcopenia  (-0.72) | -1.14e-03 | -6.07e-03,0.00 | -17.80% | -1.43,1.03 | .260 |
|  | carotenoids->METS->Sarcopenia  (-0.46) | 3.66e-03 | -2.12e-04,0.01 | 2.84% | -0.05,0.29 | .226 |
| Mexican American | Inflammation |  |  |  |  |  |
|  | retinol->ALP->Sarcopenia  (-0.68) | -7.20e-03 | -1.71e-02,0.00 | -1.20% | -0.69,0.54 | .960 |
|  | carotenoids->ALP->Sarcopenia  (-5.91) | 8.59e-03 | 5.44e-05,0.02 | 6.23% | -0.36,0.60 | .634 |
|  | Metabolism |  |  |  |  |  |
|  | retinol->METS->Sarcopenia  (-2.48) | -8.22e-03 | -1.83e-02,0.00 | -15.56% | -1.93,1.32 | .450 |
|  | carotenoids->METS->Sarcopenia  (-2.03) | 8.33e-03 | 4.65e-04,0.02 | 8.99% | -0.05,0.61 | .154 |
| Non-  Hispanic White | Inflammation |  |  |  |  |  |
|  | retinol->ALP->Sarcopenia  (-0.56) | -9.35e-04 | -1.13e-02,0.01 | -63.49% | -1.55,2.20 | .640 |
|  | carotenoids->ALP->Sarcopenia  (-4.14) | 6.13e-03 | -5.14e-04,0.01 | 28.69% | 0.10,1.12 | <2e-16 |
|  | Metabolism |  |  |  |  |  |
|  | retinol->METS->Sarcopenia  (-0.57) | -6.46e-04 | -1.10e-02,0.01 | -13.00% | -0.84,0.97 | .530 |
|  | carotenoids->METS->Sarcopenia  (0.02) | 8.60e-03 | 2.29e-03,0.02 | -0.03% | -0.04,0.10 | .746 |
| Non-  Hispanic Asian | Inflammation |  |  |  |  |  |
|  | retinol->ALP->Sarcopenia  (-0.51) | 1.40e-02 | 1.39e-03,0.03 | 2.23% | -0.14,0.28 | .672 |
|  | carotenoids->ALP->Sarcopenia  (1.57) | 4.52e-03 | -6.25e-03,0.02 | -29.41% | -2.24,2.37 | .360 |
|  | Metabolism |  |  |  |  |  |
|  | retinol->METS->Sarcopenia  (0.68) | 1.49e-02 | 1.82e-03,0.03 | -3.89% | -0.33,0.10 | .480 |
|  | carotenoids->METS->Sarcopenia  (0.67) | 4.03e-03 | -7.06e-03,0.01 | -15.32% | -1.34,2.29 | .580 |

**Supplementary Figures**


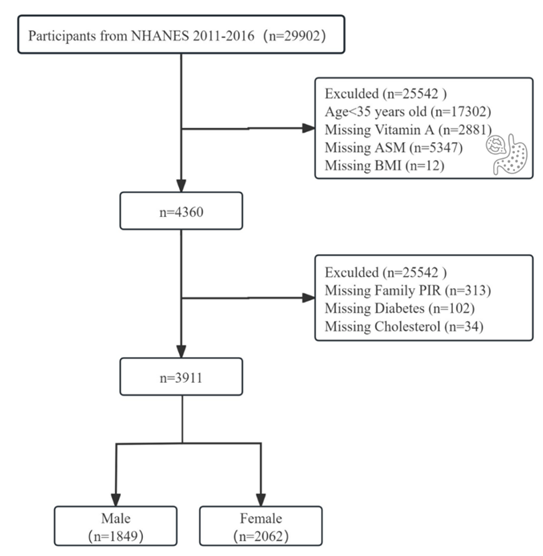


**Figure S1** Flowchart of participants included in this study.


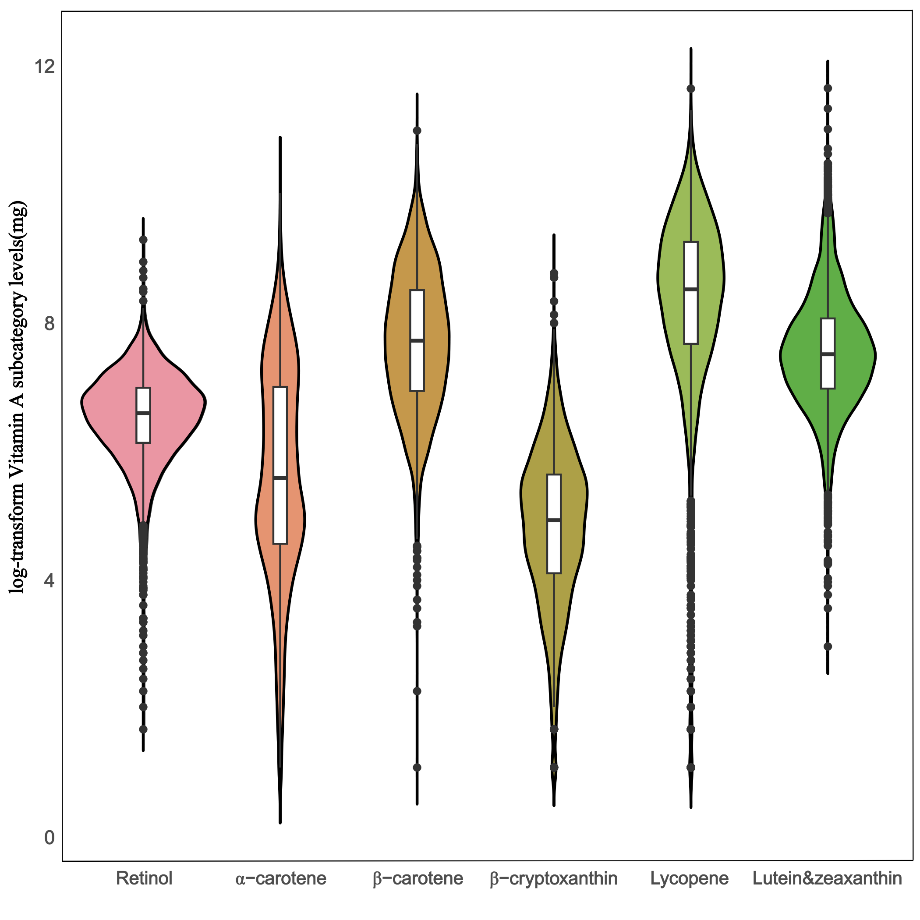


**Figure S2** Violin plots of Vitamin A intake across all participants.


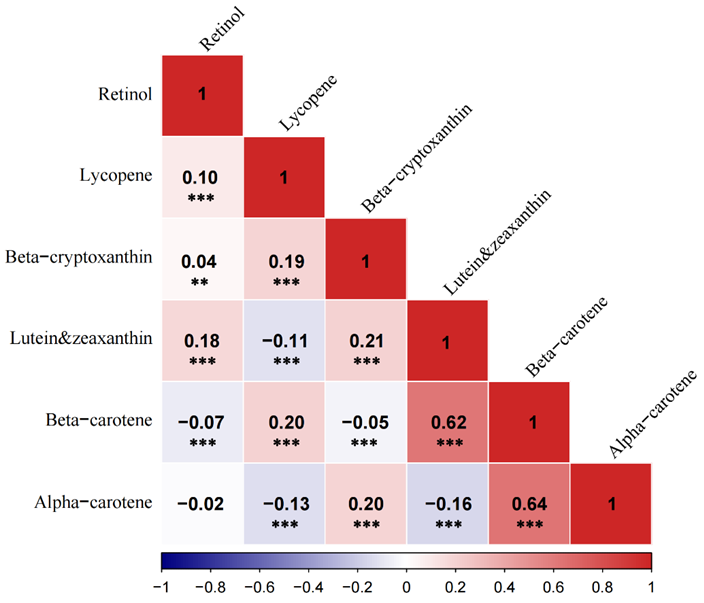


**Figure S3** Spearman’s partial correlation heatmap of retinol and six individual carotenoids. Significance: * p < 0.05, ** p < 0.01, and *** p < 0.001.


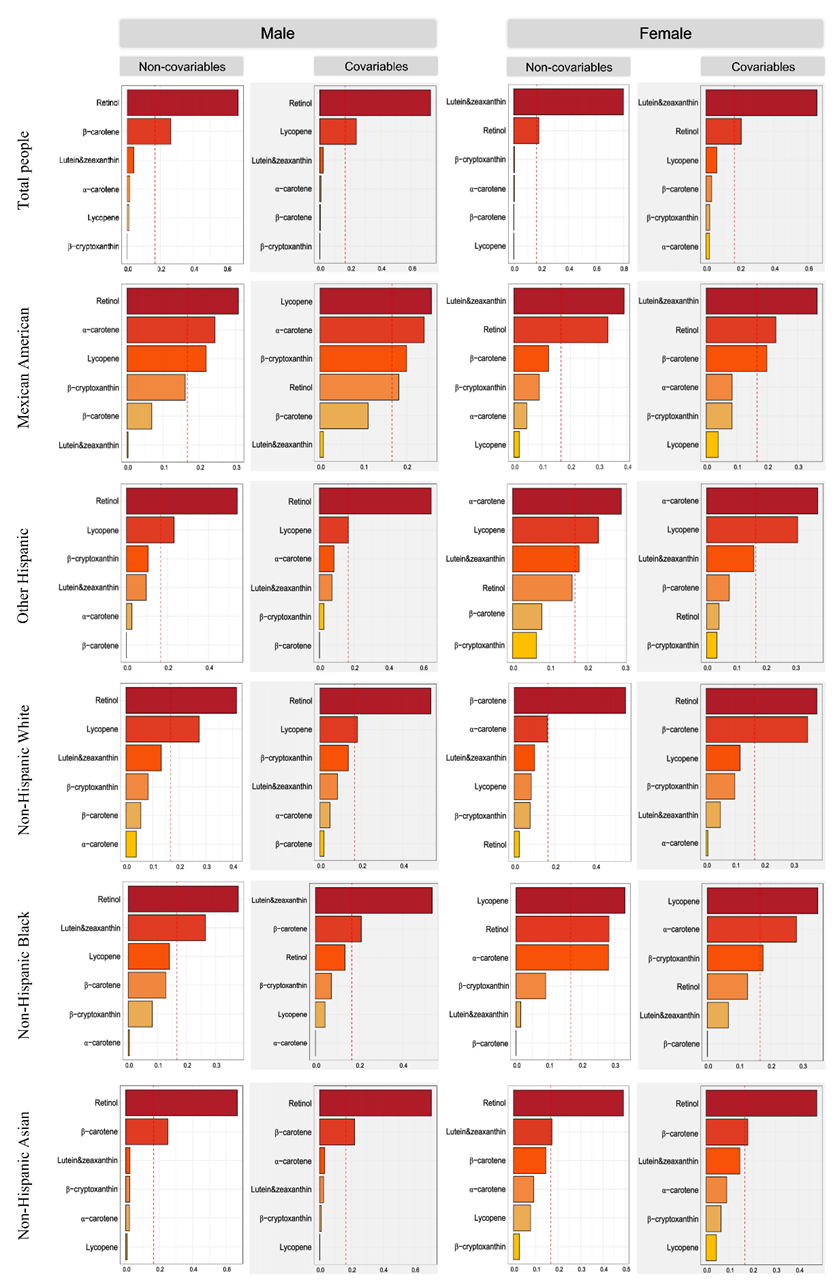


**Figure S4** Positively weighted values of retinol and six individual carotenoids for muscle mass (ASM/BMI) estimated by the WQS model.
